# Double Gallbladder Identified and Managed by Laparoscopic Cholecystectomy: A Case Report

**DOI:** 10.7759/cureus.103063

**Published:** 2026-02-05

**Authors:** Edgar A Flores García, Hector A Lopez Villicaña, José I Rodríguez Murua, Jennifer Navarro Morales, Jorge A Vazquez Tovar, Juan F Maciel Muñoz, Yessenia Escobedo Fernandez

**Affiliations:** 1 Surgery, Hospital General Nuevo Gómez Palacio, Gómez Palacio, MEX

**Keywords:** biliary anomaly, double gallbladder, duplicated cystic duct, gallbladder duplication, laparoscopic cholecystectomy

## Abstract

Gallbladder duplication is a rare congenital biliary anomaly that can pose diagnostic and surgical challenges due to anatomical variations, particularly when duplicated cystic ducts are present. We report the case of a patient who presented with recurrent right upper quadrant pain associated with cholelithiasis and was scheduled for elective laparoscopic cholecystectomy. During surgery, a duplicated gallbladder with two cystic ducts was unexpectedly identified. Intraoperative findings confirmed the anomaly, and careful dissection of Calot’s triangle allowed safe identification and ligation of the biliary structures, enabling successful resection of both gallbladders without complications. The postoperative course was uneventful, and the patient was discharged on postoperative day 1. This case underscores the importance of meticulous intraoperative assessment of biliary anatomy and demonstrates that laparoscopic management can be safe and effective in symptomatic patients, even when gallbladder duplication is not recognized preoperatively.

## Introduction

Duplication of the gallbladder is a rare congenital anomaly of the extrahepatic biliary tract, with an estimated incidence of approximately 1 in 4,000 to 5,000 individuals based on autopsy and surgical series [1,2]. This malformation originates during the fourth to sixth week of embryonic development, when abnormal budding, bifurcation, or persistence of the cystic primordium arising from the hepatic diverticulum results in the formation of two distinct gallbladder cavities [1]. In some cases, each gallbladder possesses its own independent cystic duct that drains separately into the common bile duct or other biliary structures [3,4].

The anomaly was first comprehensively studied by Boyden in 1926, who provided detailed embryological insights and described various morphological patterns in humans and comparative anatomy [1]. Gross later reviewed a large series of congenital biliary anomalies, further highlighting the clinical spectrum of duplicated gallbladders [2]. The most widely accepted classification was proposed by Harlaftis et al. [3], dividing gallbladder duplication into two main types: Type I (split primordium or vesica fellea divisa), which includes septated, V-shaped, and Y-shaped variants sharing a single cystic duct; and Type II (accessory gallbladder or vesica fellea duplex), characterized by two completely separate gallbladders with independent cystic ducts draining into the biliary tree, often referred to as the H-shaped or ductular variant [3]. Associated anatomical variations in the extrahepatic biliary tree, including anomalous cystic duct insertions, are frequently observed and contribute to the surgical complexity of these cases [3,5].

Although duplicated gallbladders are often asymptomatic and discovered incidentally during imaging studies, autopsy, or abdominal surgery, they may predispose patients to cholelithiasis, acute or chronic cholecystitis, choledocholithiasis, or biliary dyskinesia affecting one or both vesicles [6,7]. More importantly, failure to recognize this anomaly preoperatively or intraoperatively during cholecystectomy, particularly in the laparoscopic era, carries a significant risk of iatrogenic biliary injury, incomplete removal, retained calculi, or persistent symptoms due to a missed accessory gallbladder [8,9].

Herein, we present a rare case of true double gallbladder with duplicated cystic ducts, identified intraoperatively and successfully managed through laparoscopic double cholecystectomy.

## Case presentation

A 32-year-old female patient presented with symptomatic cholelithiasis. Preoperative laboratory investigations, including complete blood count, liver function tests, and coagulation profile, were within normal limits. Abdominal ultrasound revealed a gallbladder of normal size and wall thickness, with a normal caliber cystic duct and common bile duct. Multiple anechoic images with posterior acoustic shadowing were noted within the lumen, consistent with gallstones measuring approximately 0.5-1.5 cm.

**Figure 1:**
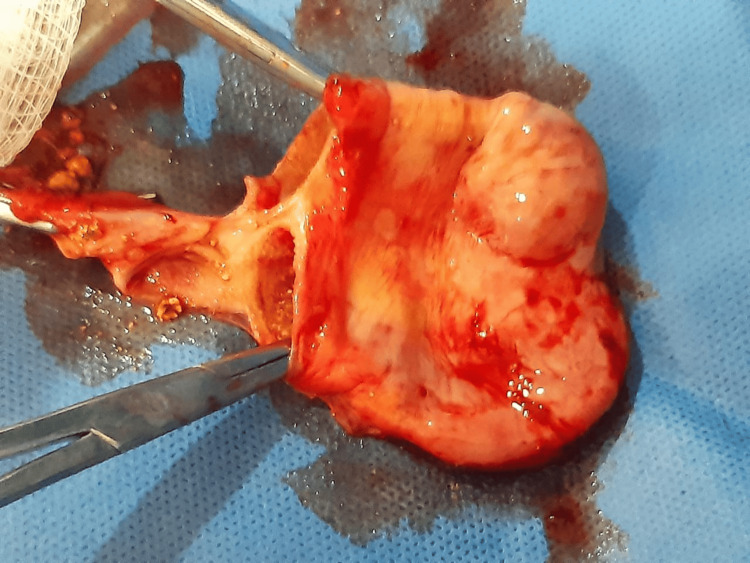
Gross photograph of the resected double gallbladder specimen, opened from the cystic duct region to expose the interior, revealing two distinct cavities separated by a longitudinal septum. This finding confirms the presence of a septate component or true duplication with independent lumens, consistent with the Type II (vesica fellea duplex) anomaly.

**Figure 2:**
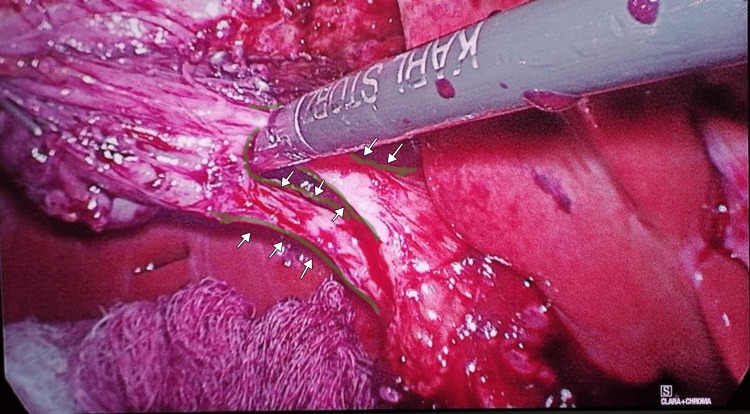
Intraoperative laparoscopic view during dissection of Calot’s triangle, demonstrating two distinct cystic ducts (highlighted in green) draining independently from the duplicated gallbladders. The structures are also indicated with white arrows to facilitate identification.

**Figure 3:**
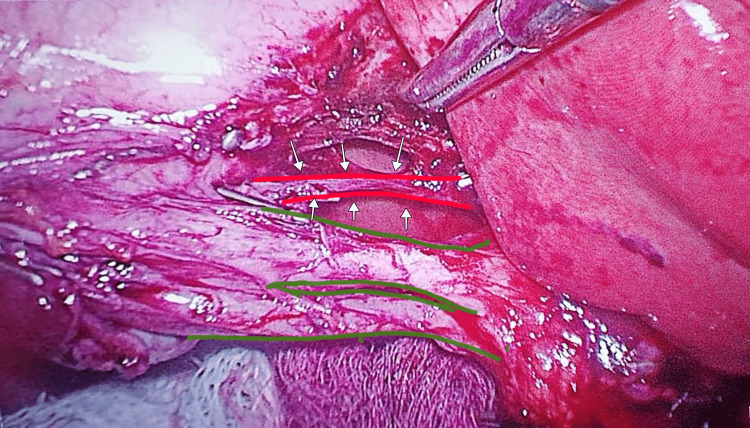
Close-up intraoperative laparoscopic view showing the duplicated cystic ducts (highlighted in green) after further dissection, with the cystic artery (highlighted in red) clearly delineated. White arrows indicate the cystic artery.

The patient underwent an elective laparoscopic cholecystectomy. Intraoperatively, a rare congenital anomaly was identified: a double gallbladder characterized by a longitudinal septum dividing the organ into two separate cavities (Figure 1), each with its own independent cystic duct inserting separately into the common bile duct (Harlaftis Type II or ductular variant) (Figures 1-3).

Multiple calculi were present, predominantly in one cavity. Careful dissection was performed, establishing the critical view of safety for each cystic structure. Both cystic ducts were clipped and divided independently, and a complete double cholecystectomy was achieved without complications or the requirement for intraoperative cholangiography.

The resected specimen was submitted for histopathological examination, which confirmed chronic cholecystitis associated with cholelithiasis, without evidence of dysplasia or malignancy. Postoperatively, the patient had an uneventful recovery, was discharged on postoperative day 1, and reported complete resolution of symptoms at follow-up.

## Discussion

Gallbladder duplication is a rare congenital anomaly of the biliary tract, with a reported incidence of approximately 1 in 4000 individuals [1]. This malformation results from aberrant budding of the hepatic diverticulum during the fifth to sixth week of embryogenesis [2]. The classification proposed by Harlaftis remains the most widely accepted system for categorizing gallbladder duplication, dividing cases into three types: Type I (split primordium group, including septate, V-shaped, or Y-shaped variants with a single cystic duct); Type II (vesica fellea duplex, characterized by two separate gallbladders with independent cystic ducts); and Type III (complex variants, such as triple gallbladders) [3]. Representative imaging examples illustrating this classification have been previously described and are available in the Radiopaedia database [4]. The present case corresponds to a Type II duplication with duplicated cystic ducts, a configuration associated with increased surgical complexity due to variations in extrahepatic biliary anatomy.

Clinically, duplicated gallbladders are frequently asymptomatic and are often detected incidentally during imaging, surgery, or autopsy [5,6]. When symptomatic, patients typically present with biliary manifestations such as cholelithiasis or cholecystitis, which are indistinguishable from those observed in patients with a single gallbladder [7]. Several reports describe cases in which gallbladder duplication with double cystic ducts was identified either preoperatively or intraoperatively, underscoring the diagnostic challenges associated with this anomaly [7,8]. Variations in biliary and vascular anatomy increase the risk of iatrogenic injury during cholecystectomy if the condition is not recognized [9]. Although preoperative diagnosis has historically been uncommon, the use of imaging modalities such as ultrasound, computed tomography, and magnetic resonance cholangiopancreatography may improve detection rates in selected cases [10,11].

Laparoscopic cholecystectomy has been reported as a feasible and safe approach for the management of gallbladder duplication, including complete removal of both gallbladders in a single procedure [6]. Successful laparoscopic management of Type II duplications has been described, with adjunctive techniques such as laparoscopic ultrasound used to confirm biliary anatomy intraoperatively [12,13]. Failure to recognize and excise an accessory gallbladder in symptomatic patients may result in persistent symptoms and the need for reoperation.

In the present case, gallbladder duplication with duplicated cystic ducts was identified intraoperatively during laparoscopic cholecystectomy. Careful and systematic dissection allowed complete resection of both gallbladders without complications or conversion to open surgery. This case contributes to the existing literature by demonstrating that, even in the absence of a preoperative radiologic diagnosis, meticulous intraoperative anatomical assessment can enable safe laparoscopic management of this rare biliary anomaly.

## Conclusions

This report describes a case of symptomatic gallbladder duplication with duplicated cystic ducts identified intraoperatively during laparoscopic cholecystectomy. Despite the absence of a preoperative radiologic diagnosis, careful dissection and systematic identification of the biliary anatomy allowed complete resection of both gallbladders and their respective cystic ducts without complications. The patient had an uneventful postoperative recovery. This case highlights the importance of intraoperative vigilance and meticulous anatomical assessment when unexpected biliary variants are encountered during laparoscopic surgery.

## References

[1] Boyden EA (1926). The accessory gallbladder - an embryological and comparative study of aberrant biliary vesicles occurring in man and the domestic mammals. Am J Anat.

[2] Gross RE (1936). Congenital anomalies of the gallbladder: a review of 148 cases, with report of a double gallbladder. Arch Surg.

[3] Harlaftis N, Gray SW, Skandalakis JE (1977). Multiple gallbladders. Surg Gynecol Obstet.

[4] Di Muzio B, Elfeky M, Thibodeau R (2026). Multiple gallbladders. Radiopaedia.

[5] Lamah M, Karanjia ND, Dickson GH (2001). Anatomical variations of the extrahepatic biliary tree: review of the world literature. Clin Anat.

[6] Desolneux G, Mucci S, Lebigot J, Arnaud JP, Hamy A (2009). Duplication of the gallbladder. A case report. Gastroenterol Res Pract.

[7] Musleh MG, Burnett H, Rajashanker B, Ammori BJ (2017). Laparoscopic double cholecystectomy for duplicated gallbladder: a case report. Int J Surg Case Rep.

[8] Poh WS, Menon T, Wijesuriya R, Misur P (2022). Duplicated gallbladder with double cystic duct: hidden in plain sight. J Surg Case Rep.

[9] Chatterjee S, Mahato SP, Nayak SD, Prasad S (2015). Gall bladder duplication - a rare anomaly detected on preoperative imaging. Hellenic J Surg.

[10] Gigot J, Van Beers B, Goncette L (1997). Laparoscopic treatment of gallbladder duplication. Surg Endosc.

[11] Kim RD, Zendejas I, Velopulos C (2009). Duplicate gallbladder arising from the left hepatic duct: report of a case. Surg Today.

[12] Ozgen A, Akata D, Arat A, Demirkazik FB, Ozmen MN, Akhan O (1999). Gallbladder duplication: imaging findings and differential considerations. Abdom Imaging.

[13] Paraskevas GK, Raikos A, Loannidis O, Papaziogas B (2011). Duplicated gallbladder: surgical application and review of the literature. Ital J Anat Embryol.

